# The Liver as a Target Organ for Gene Therapy: State of the Art, Challenges, and Future Perspectives

**DOI:** 10.3390/ph5121372

**Published:** 2012-12-10

**Authors:** Frank Jacobs, Stephanie C. Gordts, Ilayaraja Muthuramu, Bart De Geest

**Affiliations:** Centre for Molecular and Vascular Biology, Department of Cardiovascular Sciences, Catholic University of Leuven, Campus Gasthuisberg, Herestraat 49, B-3000 Leuven, Belgium; E-Mails: jacobsfrank@gmail.com (F.J.); stephanie.gordts@med.kuleuven.be (S.C.G.); ilayaraja.muthuramu@med.kuleuven.be (I.M.)

**Keywords:** gene transfer, liver, hepatocytes, fenestrae, AAV

## Abstract

The liver is a target for gene therapy of inborn errors of metabolism, of hemophilia, and of acquired diseases such as liver cancer and hepatitis. The ideal gene transfer strategy should deliver the transgene DNA to parenchymal liver cells with accuracy and precision in the absence of side effects. Liver sinusoids are highly specialized capillaries with a particular endothelial lining: the endothelium contains open fenestrae, whereas a basal lamina is lacking. Fenestrae provide a direct access of gene transfer vectors to the space of Disse, in which numerous microvilli from parenchymal liver cells protrude. The small diameter of fenestrae in humans constitutes an anatomical barrier for most gene transfer vectors with the exception of adeno-associated viral (AAV) vectors. Recent studies have demonstrated the superiority of novel AAV serotypes for hepatocyte-directed gene transfer applications based on enhanced transduction, reduced prevalence of neutralizing antibodies, and diminished capsid immune responses. In a landmark clinical trial, hemophilia B was successfully treated with an AAV8 human factor IX expressing vector. Notwithstanding significant progress, clinical experience with these technologies remains very limited and many unanswered questions warrant further study. Therefore, the field should continue to progress as it has over the past decade, cautiously and diligently.

## 1. Hepatocyte-Directed Gene Transfer

The liver is a central organ in metabolism. Consequently, numerous inherited metabolic disorders have their origin in this organ. Inborn errors of metabolism may lead to accumulation of toxic products in hepatocytes and extensive hepatotoxicity, as observed in disorders like α_1_-antitrypsin deficiency, type I tyrosinemia, or Wilson disease [1]. In other metabolic diseases, such as in Crigler-Najjar syndrome type I, ornithine transcarbamylase deficiency, familial hypercholesterolemia, and hemophilia A and B, manifestations are primarily extrahepatic [1]. The liver is a target for gene therapy of inborn errors of metabolism, of hemophilia, and of acquired diseases such as liver cancer and hepatitis [2,3]. More precisely, the real target for hepatocyte-directed gene transfer strategies are the parenchymal liver cells or hepatocytes *stricto sensu*. The ideal gene transfer strategy should target these cells with accuracy and precision in the absence of any untoward effects.

One salient difference between gene therapy and therapies based on administration of small chemical compounds or of monoclonal antibodies is the size of the therapeutic agent in gene transfer applications. We will discuss the strategic importance of this point for gene transfer development in the next two sections.

## 2. Parenchymal Liver Cells as a Gene Transfer Target: The Role of Sinusoidal Cells

Liver sinusoids are highly specialized capillaries with a particular endothelial lining: the thin endothelium contains open fenestrae, whereas a basal lamina is lacking [4]. Parenchymal liver cells comprise approximately 67% of resident liver cells whereas sinusoidal cells comprise the remaining cells [5,6,7]. Besides endothelial cells, sinusoidal cells comprise Kupffer cells (resident liver macrophages), fat-storing cells (also called stellate cells or Ito cells), and pit cells (natural killer cells). Liver sinusoidal endothelial cells constitute 70%, Kupffer cells 20%, stellate cells 10%, and pit cells less than 1% of the number of sinusoidal cells [5,7,8]. Kupffer cells and liver sinusoidal endothelial cells make up the reticulo-endothelial cells of the liver. Kupffer cells account for 80% to 90% of resident macrophages in the entire body [9]. Sinusoidal endothelial cells are scavenger cells that are able to internalize particles up to 0.23 µm under physiologic conditions *in vivo* [10]*.* Larger particles are taken up by Kupffer cells [10]. Since most gene transfer vectors have a diameter below 0.23 µm, uptake of vectors by both Kupffer cells and liver sinusoidal endothelial cells is a serious obstacle that limits the efficiency of hepatocyte-directed gene transfer [11,12,13,14,15,16,17,18]. 

Most experimental work on the role of liver reticulo-endothelial cells in relation to hepatocyte transduction has been performed in adenoviral gene transfer studies. Several investigations have demonstrated that different adenoviral serotypes are rapidly sequestered in the liver after intravenous delivery [19,20,21]. Cellular uptake of adenoviral vectors after systemic gene transfer occurs predominantly in non-parenchymal liver cells (*i.e.* mainly liver sinusoidal endothelial cells and Kupffer cells) [11,12,13,14]. We have demonstrated that uptake of vectors by non-parenchymal liver cells (*i.e.* mainly liver sinusoidal endothelial cells and Kupffer cells) inversely correlates with transduction of parenchymal liver cells [14] and is mouse strain-dependent. The transgene DNA copy number in the non-parenchymal liver cells at one hour after transfer in Balb/c mice was nearly 6-fold higher than in C57BL/6 mice [14]. This difference in scavenging of vectors between both strains is a major determinant of the approximately 3-fold higher transgene DNA levels and higher transgene expression levels in parenchymal liver cells of C57BL/6 mice compared to Balb/c mice [14]. 

Further evidence for a major role of liver reticulo-endothelial cells as a determinant of hepatocyte transduction comes from experiments with clodronate liposomes. Depletion of Kupffer cells and macrophages in the liver by intravenous administration of clodronate liposomes results in significantly increased transgene DNA levels in parenchymal liver cells [14] and in increased transgene expression [13,14,22,23]. Since liver sinusoidal endothelial cell function may be modified by Kupffer cells [24,25], it cannot be excluded that part of the effect of clodronate liposomes is due to reduced activation of liver sinusoidal endothelial cells by Kupffer cells. Besides clodronate liposomes, pre-administration of polyinosinic acid, a scavenger receptor A ligand, before gene transfer has been shown to prevent sequestration of adenoviral vectors in Kupffer cells and to enhance parenchymal liver cell transduction [26]. Transient saturation of the reticulo-endothelial system with phosphatidylcholine liposomes or with Intralipid^® ^also reduces uptake of vectors in non-parenchymal liver cells and augments hepatocyte transduction [14]. Taken together, interventions that result in decreased uptake of adenoviral vectors in liver reticulo-endothelial cells consistently augment hepatocyte transduction. 

## 3. Parenchymal Liver cells as a Gene Transfer Target: the Role of Sinusoidal Fenestrae

Fenestrae are clustered in sieve plates and provide an open pathway between the sinusoidal lumen and the space of Disse, in which numerous microvilli from parenchymal liver cells protrude [4,27]. Whereas the Kupffer cells and liver sinusoidal endothelial cells constitute a barrier for access to the parenchymal liver cells, sinusoidal fenestrae form an escape route to the space of Disse and the microvillous surface of hepatocytes. Sinusoidal fenestrae have no diaphragm. Although fenestrae constitute an open communication between the sinusoidal lumen and the space of Disse, they will act as a sieve and will mechanically restrict the transendothelial transport of gene transfer vectors according to their size.

Fenestrae measure between 100 nm and 200 nm and significant species differences in their size exist [4,27,28,29,30,31]. Using state of the art transmission electron microscopy measurements, we have previously demonstrated that the average diameter of fenestrae is significantly larger in Dutch Belt rabbits (124 nm) [32], in Sprague Dawley rats (150 nm in the pericentral area and 175 nm in the periportal area) [28], and in C57BL/6 mice (141 nm) [33] than in humans with a healthy liver (107 nm) [34]. In contrast, the average diameter in New Zealand White rabbits (103 nm) [33] and Fauve de Bourgogne rabbits (105 nm) [32] was similar to humans. Importantly, interindividual variation of the average diameter of fenestrae within the same species or strain is low, as indicated by coefficients of variation between 3%–8%. However, within the same animal or individual, there is a Gaussian distribution of diameters around the average with skewing to the right [29]. 

**Figure 1:**
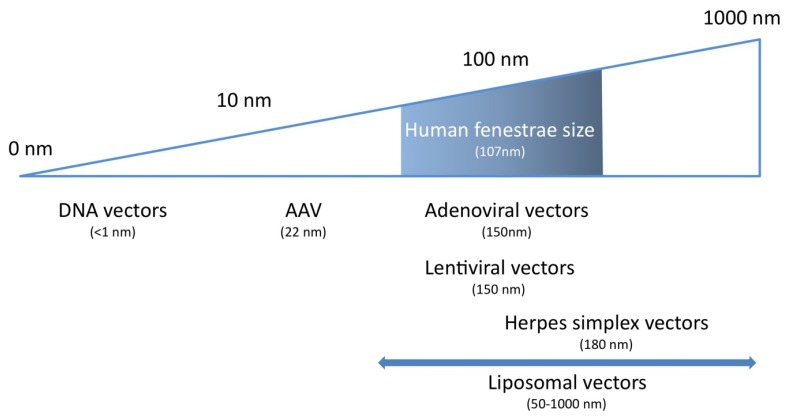
Relevance of gene therapy vector dimensions for gene transfer efficiency into hepatocytes. Human liver sinusoidal fenestrae show a Gaussian size distribution (shaded box) and have an average diameter of 107 nm. To ensure efficient transendothelial passage of gene transfer vectors, their size should be small enough to pass through the fenestrae into the space of Disse. The only gene therapy systems that display this highly desirable feature are naked DNA transfer and AAV-mediated transfer.

Before summarizing experimental evidence that these diameters are a determinant of gene transfer efficiency into hepatocytes, a discussion of the diameter of gene transfer vectors is warranted. Using cryo-electron microscopy, adenoviral serotype 5 virions were shown to have a diameter of 93 nm with protruding fibers of 30 nm [33], whereas the diameter of a vesicular stomatitis virus-G pseudotyped human immunodeficiency virus-1 derived lentiviral vector was found to be 150 nm [33]. Adeno-associated viral (AAV) serotype 2 vectors have an average diameter of 22 nm [35] and this size does not vary significantly between different serotypes of AAV vectors [36,37,38,39]. Herpes simplex virions have been reported to be as large as 180 nm [40]. The diameter of liposomes used for non-viral gene transfer varies between 50 nm and 1000 nm and is highly dependent on production parameters [41,42]. All in all, from a theoretical point of view, it is clear that fenestrae in humans may act as a sieve for most vectors with the obvious exception of AAV vectors (Figure 1).

The experimental evidence for the role of the diameter of fenestrae in hepatocyte transduction by adenoviral vectors is in essence based on two seminal observations [32,33]. Firstly, the ratio of transgene DNA in parenchymal liver cells *versus* transgene DNA in non-parenchymal liver cells early after gene transfer correlates directly with the diameter of fenestrae [33]. Secondly, interventions that increase the diameter of fenestrae induce enhanced transgene DNA uptake in hepatocytes and augmented transgene expression [32,33].

The diameter of fenestrae in New Zealand White rabbits and Fauve de Bourgogne rabbits resembles the size in humans. Adenoviral gene transfer into hepatocytes is highly inefficient in Fauve de Bourgogne rabbits and New Zealand White rabbits [32,33]. Therefore, one would predict a similar low efficiency in humans. Evidence supporting this hypothesis comes from the ornithine transcarbamylase deficiency trial, which remains the only hepatocyte-directed clinical trial in humans using adenoviral vectors [43]. Levels of transgene expression in this trial were indeed much lower than what would have been predicted based on preclinical animal models [43].

## 4. Characteristics of the Ideal Vector for Hepatocyte-Directed Gene Transfer

Table 1 summarizes the characteristics of the ideal vector for hepatocyte-directed gene transfer. Based on the discussion in the previous two sections, a *conditio sine qua non* for efficient hepatocyte-directed gene transfer is facilitated passage of vectors through fenestrae. Therefore, this criterion is listed on top in the Table 1. Considering that the diameter of fenestrae in humans with a healthy liver is 107 nm [34], only AAV vectors and some non-viral gene transfer vectors fulfil this criterion in humans. The second criterion implies efficient transduction or transfection of hepatocytes. A vector not only has to pass the fenestrae but the transgene DNA must be subsequently delivered from the extracellular space of Disse into the nucleus of hepatocytes with high efficiency. This requires efficient cellular uptake and efficient nuclear delivery without annihilating intracellular degradation of transgene DNA. Using these first two criteria as decisive parameters for selection of the ideal gene transfer vector system, AAV vector technology remains the only valid option at present for hepatocyte-directed gene transfer in humans. However, one non-viral gene transfer strategy has been demonstrated to result in transfection of a high percentage of hepatocytes in mice. This method is hydrodynamic gene transfer. Considering the theoretically high potential of this technology, a more detailed discussion of this technology is indicated.

**Table 1:** Characteristics of the ideal vector for hepatocyte-directed gene transfer.

Properties	Vector
Facilitated passage through fenestrae	AAV, some non-viral gene transfer vectors
Efficient transduction or transfection	AAV, adenoviral vectors
Target specific	No vector system, apply transcriptional targeting
Repeated use possible	Minicircles, non-viral gene transfer vectors, apply serotype switch
No or minor vector-induced innate immune responses	AAV, minicircles?
No or minor vector-induced adaptive immune responses	Minicircles, non-viral gene transfer vectors
No non-immune toxicity	AAV, some non-viral gene transfer vectors
High insertion capacity	Adenoviral vectors, minicircles, non-viral gene transfer systems

In 1999, Liu *et al.* [44] and Zhang *et al.* [45] independently demonstrated that hydrodynamic gene transfer method results in high transgene expression in the liver that is in a similar order of magnitude as levels obtained after adenoviral or AAV gene transfer. This non-viral gene transfer method in mice entails the injection of a large bolus of a physiological solution, equivalent to 8 to 10% of the body weight, into the tail vein within a period of 5 to 7 seconds. The subsequent right heart volume overload results in a retrograde flow through the *vena cava* and in particular in a retrograde flow into the hepatic veins. Consequently, intrahepatic pressure increases and the DNA containing solution is forced out of the hepatic sinusoids into the parenchymal cells of the liver. Following systemic hydrodynamic gene transfer in mice and rats, more than 90% of the injected DNA can be retrieved in the liver [46]. In addition, microscopic analysis has indicated that transduced hepatocytes are predominantly located in the pericentral region [47]. This predilection may be explained by the fact that sinusoids are wider and straighter and contain more fenestrae per unit of surface in the pericentral area than in the periportal area [46,48].

Hydrodynamic gene transfer of minicircles may result in persistent transgene expression in mice [49,50,51]. Minicircles are devoid of bacterial sequences that cause transcriptional silencing [49,50]. In addition, repeated administration of minicircle DNA is possible. Readministration may be required since episomal DNA instability in the long-term cannot be excluded. Notwithstanding the attractive features of hydrodynamic gene transfer, application of this technology in larger species is cumbersome. The degree of systemic volume overload as applied in rodents obviously cannot be extrapolated to larger species. Therefore, great efforts have been made to develop innovative hydrodynamic gene transfer techniques that require lower volumes. Zhang *et al.* [52] demonstrated that, using localized hydrodynamic gene transfer via the hepatic vein in rats, the required volume for injection could be reduced to less than 2% of the body weight. Eastman *et al.* [53] showed that in rabbits a volume of 15 ml/kg (*i.e.* 1.5% of the body weight) suffices to successfully transduce hepatocytes when using catheter-based isolation of the liver. However, levels of transgene expression were 10- to 20-fold lower compared to mice [53]. Yoshino *et al.* [54], Alino *et al.* [55], and Fabre *et al.* [56] reported hydrodynamic gene transfer into pig liver or isolated liver segments using catheterization techniques. These procedures were well tolerated and resulted in intrahepatic vascular pressures up to 4-fold higher than observed in mice and rats. Nonetheless, levels of transgene expression were 200-fold [54,56] to 10 000-fold [55] lower following hydrodynamic gene transfer in pigs when compared to mice. Suda *et al.* [57] developed a computer-assisted hydrodynamic gene transfer device (a so-called ‘hydrojector’), wherein all injection parameters (volume, speed, …) are coordinated via an intrahepatic pressure sensor. This technology was applied in mice, rats, and pigs, but again, large species differences in efficiency were observed. The consistent failure of hydrodynamic gene transfer in larger species may be due to anatomical differences of the liver, in particular differences in hepatic compliance and differences in the structure of the hepatic sinusoids. 

The third criterion, absolute target specificity, is not met by any vector system. However, transgene expression can be restricted to hepatocytes by use of hepatocyte-specific expression cassettes [58,59,60,61,62,63]. This transcriptional targeting has been shown to be a successful strategy to avoid the development of a humoral immune response against a potentially immunogenic transgene product in several studies [58,59,60,61,62,64,65]. From an immunological point of view, the liver is a particular gene transfer target. It has been described as a tolerogenic organ more than 40 years ago [66]. Naive T cells encounter liver antigens initially in the liver, rather than in lymphoid tissue [67]. Whereas lymph nodes and the spleen are anatomical compartments that provide a particular microarchitecture and microenvironment for the induction of immunity, antigen presentation in the liver takes places in a completely different setting. The liver microenvironment determines the tolerogenic properties of liver dendritic cells [68,69]. This is a key aspect of the hepatic adaptive immune tolerance induction [70]. 

Repeated administration (criterion 4) is possible in case of non-viral gene transfer vectors and in case of hydrodynamic gene transfer of naked DNA. Readministration is not possible for viral vectors but this problem may be circumvented by injection of a vector of a different serotype [71,72]. The requirement for readministration after AAV-mediated gene transfer will depend on the long-term episomal transgene DNA stability. In general, this stability is remarkably good for AAV episomes.

In contrast to potentially very serious innate immune responses after adenoviral gene transfer [43,73,74,75], AAV gene transfer induces very weak or absent innate immune responses to viral capsids both in mice [76] and monkeys [77]. Serum levels of five major inflammatory cytokines (tumor necrosis factor-α, interferon-γ, interleukin-6, interleukin-10, and interleukin-12) were not elevated in macaques after gene transfer with vectors based on AAV serotypes 2, 7, and 8 [77]. Although non-viral gene transfer systems could theoretically be devoid of innate immune responses, serious innate immunity may occur [78,79,80], which is not only related to the presence of unmethylated CpG motifs. Further criteria of the ideal vector will be discussed within the framework of the next sections on AAV-mediated hepatocyte-directed gene transfer.

## 5. AAV-Mediated Hepatocyte-Directed Gene Transfer: Introduction

AAV are small, nonenveloped, single-stranded DNA viruses and belong to the *Dependovirus* genus of the *Parvoviridae* family. Up to date, 11 different serotypes comprising over 120 capsid variants have been isolated from humans, nonhuman primates, cows, dogs, horses, and birds [81,82,83,84,85]. Compared to the genome size and structure of other viral vectors, the AAV genome is relatively small and simple, at approximately 4.7 kb, and contains only two genes, *rep* and *cap*, that encode four Rep proteins and three capsid proteins, flanked by two 145-base inverted terminal repeats (ITRs). Recombinant and replication defective AAV vectors used for gene transfer applications retain only the ITRs, which are necessary for replication, packaging, and integration, while all viral coding sequences are entirely removed [86]. In light of the AAV genome size, the insertion capacity of AAV vectors is clearly limited.

Several favorable features have made AAV a valid candidate for hepatocyte-directed gene transfer. Most importantly, AAV have not been associated with human disease. Moreover, AAV are naturally replication defective and require co-infection with a helper virus, such as adenovirus or herpes virus, in order to replicate. Furthermore, AAV transduce both dividing and non-dividing cells such as hepatocytes and have been shown to maintain long-term expression in hepatocytes in the absence of chromosomal integration [80]. Whereas wild-type AAV possess the ability for site-specific integration into the human genome, this feature is absent from recombinant AAV vectors and integration into the host genome is random but relatively infrequent [87]. As already discussed, the small diameter of AAV vectors [35] is important for facilitated passage through fenestrae. 

## 6. AAV2-Mediated Hepatocyte-Directed Gene Transfer

Systemic liver-directed AAV serotype 2 mediated gene transfer resulted in persistent therapeutic factor IX (FIX) expression in immunocompetent mice, dogs, and nonhuman primates [88,89,90]. Complete correction of the bleeding diathesis in inhibitor-prone hemophilia dogs with a complete deficiency of FIX has been achieved after AAV2 gene transfer for more than 9 years [89]. Of note, onset of transgene expression following AAV2 gene transfer to the liver is slow, with a lag phase of 4 to 6 weeks, followed by a gradual increase over a period of 4-10 weeks before reaching a stabile plateau. Long-term expression in the liver has been shown to be attributable to episomally maintained vector genomes that mostly exist in the nucleus in the form of high-molecular concatamers [91,92]. 

The first clinical trial for hepatocyte-directed AAV2 gene transfer [93] was an open-label, dose-escalation study of a human FIX expressing vector delivered through the hepatic artery in patients with severe hemophilia B. One subject in the highest dose cohort receiving 2 x 10^12^ vector genomes/kg showed a therapeutic increase in FIX activity of up to 10%–12% of normal levels. In contrast to long-lasting expression in hemophilic dogs and non-human primates using similar vectors, expression at therapeutic levels in this subject was short lived. Starting at 5 weeks after vector administration, FIX gradually declined to baseline at 10 weeks post vector infusion. This decline coincided with a rise in transaminase levels between 4 and 8 weeks after vector administration. Antibodies directed against the human FIX were undetectable and no toxic or infectious causes for the increase in liver enzymes were found. Another subject that was dosed at a 5-fold lower dose also displayed a self-limited rise in liver enzymes with similar kinetics. A series of follow-up experiments suggested that T cell–mediated immunity to AAV capsid antigens may have induced destruction of AAV2–transduced hepatocytes [93,94]. However, this remains a matter of debate.

Overall, despite initial success in pre-clinical studies, several limitations of AAV2 vectors have emerged including limited transduction efficiency of hepatocytes, delayed onset of transgene expression [95], high prevalence of neutralizing antibodies in humans, and potentially destructive T-cell responses to capsids. These observations have underscored the need for further refinement of AAV vectors.

## 7. Hepatocyte-Directed Gene Transfer Using Alternative AAV Serotypes

In the past decade, the Wilson lab has spearheaded the identification and characterization of novel AAV serotypes [81,82]. Recent studies have demonstrated the superiority of these novel serotypes for gene transfer applications based on enhanced hepatocyte transduction, reduced prevalence of neutralizing antibodies, and diminished capsid immune responses [96,97,98]. The most salient increase in liver transduction and transgene expression has been observed for AAV8 and AAV9 [99,100]. In general, transgene expression levels following intravenous injection mice are 10 to 100-fold higher compared to AAV2 [82]. However, the observed advantages of AAV8 over AAV2 seem to be reduced in larger animal species such as dogs and nonhuman primates [77,101,102]. 

Mechanisms underlying the distinct biological performance of different AAV serotypes have been attributed to differences in viral entry, trafficking, uncoating, and genome processing [103,104]. Cunningham *et al.* showed that following systemic administration of AAV8 to the liver, all dsAAV8 genomes are formed within 3 days [105]. In contrast, AAV2 shows a gradual increase of dsAAV2 genomes over a period of several weeks [91]. Nakai *et al.* demonstrated that up to 90% hepatocyte transduction could be achieved after systemic AAV8-mediated gene transfer [95]. AAV8-mediated gene transfer has also been proven to be more potent in inducing immune tolerance for the transgene product [106]. 

Nathwani * et al.* [107] performed a landmark study that demonstrated for the first time long-term expression of a transgene product following systemic gene transfer to the liver at therapeutic levels in humans. In this study [107], hemophilia B was successfully treated with an AAV8 human FIX expressing vector. The vector genome was designed as a self-complementary vector in order to bypass the synthesis of a second DNA strain following hepatocyte transduction [108]. The authors also applied codon optimalisation of the transgene cDNA sequence in order to improve the efficiency of translation [109]. A dose-escalation study in six patients with severe hemophilia B was performed and patients have been followed 6-16 months [107]. Interestingly, all patients showed a therapeutic response, which was roughly dose-dependent. Human FIX levels ranged from 2%–11% of physiological levels in normal subjects. Four out of six patients discontinued prophylactic FIX treatment and remained free of spontaneous hemorrhages. The remaining two patients that remained on FIX protein therapy were able to extend the time interval between prophylactic injections. Intriguingly, one of the two patients that had received the highest vector dose displayed an asymptomatic increase in serum transaminases, which coincided with the presence of AAV8-capsid specific T cells in the peripheral blood. The other patient in the same cohort also displayed a slight increase in liver enzyme levels, although the cause hereof was less clear. Both patients received a short course of glucocorticoid therapy. Following administration of glucocorticoids, serum transaminase levels quickly returned to normal and FIX expression levels remained in the range of 3%–11% of normal. The gene transfer vector production costs for this study have been estimated at $30,000 for a patient treated with the middle dose of vector. In light of the annual costs of protein infusion therapy, it is clear that dramatic cost reductions may be achieved by widespread use of this therapy. If further studies indicate that this approach is safe, it may eventually replace the cumbersome and expensive prophylactic FIX therapy. Moreover, the use of similar vectors may spark significant progress in gene therapy for other disorders, including familial hypercholesterolemia [110], lysosomal storage diseases, and urea cycle defects [111].

## 8. Challenges for AAV-Mediated Hepatocyte-Directed Gene Transfer

**Figure 2:**
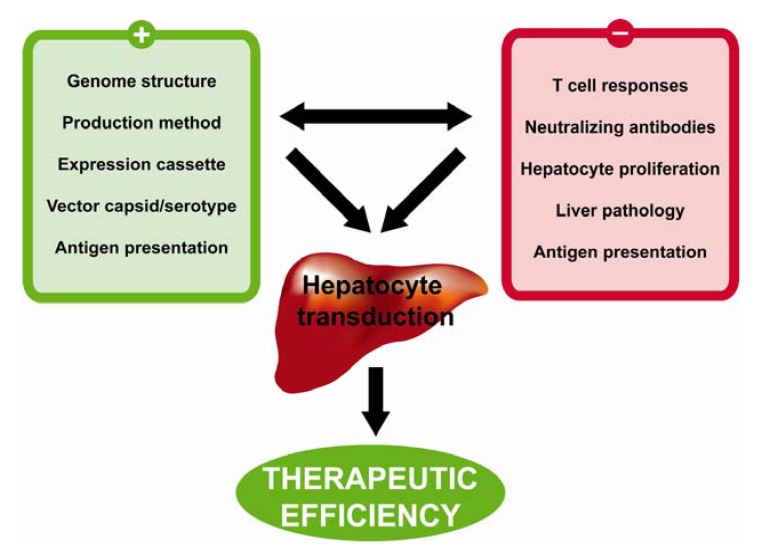
Key determinants of efficient AAV-mediated hepatocyte-directed gene transfer and of persistent transgene expression in parenchymal liver cells. Efficient gene transfer and the maintenance of persistent transgene expression in hepatocytes are influenced by many factors in both positive and negative ways. Pre-existing neutralizing antibody titers in excess of 1:10 will greatly affect hepatocyte transduction by AAV vectors. In addition, depending on the context, transduction of antigen presenting cells may contribute to the generation of cytotoxic T cells, resulting in the loss of transgene expression over time. Pathological sequelae of the liver, such as inflammation or pseudocappilarization, may also interfere with hepatocyte transduction or contribute to the priming of reactive T cells. Antigen presentation in the microenvironment of the liver plays a critical role in the generation of a tolerogenic state and may induce tolerance for the transgene product. On the other hand, hepatocyte destruction by cytotoxic T cells might occur as a consequence of MHCI presentation of capsid epitopes. In pediatric patients, fast liver growth will dilute vector genomes, resulting in a quick decline of transgene expression. Finally, different facets of vector design will greatly influence the outcome of hepatocyte-directed gene transfer strategies. Several capsid variants have shown increased hepatotropsim when compared to AAV2. Moreover, the purity and quality of AAV vector preps should be carefully monitored to ensure reproducibility and efficiency. With regard to vector expression cassette design, several studies have underscored the importance of promoter selection, optimization of coding sequences (codon optimization, Kozak sequence), intron and polyadenylation signal selection, and vector genome structure (*i.e.* scAAV vectors *versus* ssAAV vectors).

Key determinants of efficient AAV-mediated hepatocyte-directed gene transfer and of persistent transgene expression in parenchymal liver cells are summarized in Figure 2. As already mentioned, if major histocompatibility complex 1 presentation of AAV epitopes on hepatocytes occurs *in vivo*, this might be a cause of destruction of hepatocytes by cytotoxic T cells and of decline of transgene expression. Interindividual differences in antigen processing and presentation may influence the type and magnitude of the immunological response against the AAV particles [112]. Furthermore, pre-exposure to natural AAV infections may vary highly between patients, resulting in a completely different repertoire of memory T cells.

Seroneutralisation of AAV vectors in patients with prior exposure to AAV viruses is a serious obstacle for a successful outcome of the treatment. Prior exposure in mice, dogs, nonhuman primates, and humans results in the formation of complex profiles of serum antibodies capable of binding and neutralizing specific AAV serotypes [96,113]. Although human data on the effects of neutralizing antibodies on hepatocyte transduction are scarce, the absence of efficient transduction in at least one patient of the FIX clinical trial has been attributed to the higher level of neutralizing antibodies [93]. A large proportion of the human population has been exposed to AAV, with at least one-third having neutralizing antibodies against AAV2 [114]. In a recent study by the Wilson group, the authors analyzed serum samples from 888 persons, spanning four continents and 10 different countries, for the presence of neutralizing antibodies directed against a wide array of AAV serotypes [96]. In most regions, the prevalence of neutralizing antibody titers exceeding 1:10 was lowest for AAV7 (15%–30%) and AAV8 (15%–30%). In contrast, the prevalence of neutralizing antibodies against AAV1 (20%–45%) and AAV2 (30%–60%) was significantly higher. These numbers are in accordance with a recent study by Boutin *et al.*, showing the prevalence of AAV-binding Ab against AAV1 (51%) and AAV2 (59%) that were significantly higher than those for AAV5 (3.2%), AAV6 (37%), AAV8 (19%), and AAV9 (36%) [115]. The observed differences in seroprevalence against distinct serotypes most likely reflect their natural host tropism. Humans are not commonly pre-exposed to serotypes derived from nonhuman primates, resulting in lower titers of neutralizing antibodies. Finally, a significant degree of coprevalence of neutralizing antibodies against different serotypes has been observed. 

Pre-exposure to AAV has been mimicked by readministration studies in animal models. However, neutralizing antibodies generated in this context are significantly higher than could be expected following natural exposure to AAV in humans. Additionally, since neutralizing antibodies are generated in the absence of co-infection of the helper virus, they may vary in their affinity and/or breadth towards AAV. Scallan *et al.* used a model of passive transfer of pooled human IgG in mice injected with AAV2 to overcome these limitations and showed that neutralizing antibodies titers as low as 1:10 could efficiently block hepatocyte transduction in mice [116]. Importantly, sero-neutralization in this model could be circumvented by administration of vectors of serotypes AAV6 and AAV8. In this respect, AAV8 gene transfer also resulted in sustained transgene expression in dogs that had previously been treated with an AAV2 vector [102]. In light of these results, routine screening of neutralizing antibody titers in larger animals such as nonhuman primates has become general practice in preclinical studies over the past years. Moreover, the absence of detectable levels of neutralizing antibodies towards a given AAV serotype is commonly included in the criteria of eligibility for human patients participating in phase I clinical trials.

A particular concern for hepatocyte-directed gene transfer in pediatric patients is the impact of liver growth on transgene DNA persistence. Most AAV genomes reside within the nucleus in an episomal form. Consequently, transgene DNA is lost during mitosis when the nuclear envelope is degraded. In the adult mouse liver, individual hepatocytes are slowly-dividing, completing a single population turnover every 180–400 days [117]. A rapid decline of transgene expression has been observed in the context of extensive hepatocyte proliferation following partial hepatectomy in adult mice [92] or following AAV administration into neonatal mice [105,118]. Interestingly, when vector is delivered at serially higher ages up to adulthood, transgene expression is progressively more durable, correlating with the deceleration of liver growth [105]. Taken together, these studies indicate that proliferation of hepatocytes results in a nearly complete clearance of AAV genomes from the liver within two population doublings [92,105,118,119]. In humans, it is estimated that a first doubling of the liver mass occurs within 9 months following birth followed by a second doubling in mass taking up to 3 to 4 years [120]. Thus, vector readministration is likely to be necessary. This may be achieved by using distinct AAV serotypes to circumvent pre-existing immunity [102,121,122].

Another challenge is to scale-up AAV production and purification. Several innovative production systems have recently been developed that are compatible with large-scale production for clinical application [123,124,125]. These new systems use adenovirus, herpesvirus, and baculovirus hybrids to deliver the rAAV genome and *trans*-acting helper functions to the producer cells. However, whereas these systems are particularly suited for production of clinical grade candidate vectors, the need to make the hybrid viruses for each vector makes them less suited to early development and preclinical studies in which several combinations of transgene and vector serotype may need to be evaluated. 

Interestingly, Vandenberghe *et al.* recently reported a novel AAV isolation method that is based on the significant release of AAV from the cells during vector production [126,127]. The option of harvesting from supernatants rather than cell lysates should greatly simplify downstream processing of AAV vectors for research and clinical applications. In addition, using this system most AAV serotypes can be produced in the absence of serum, thus limiting concerns about possible contamination of AAV vector preparations. 

Irrespective of the modus of production, several studies have identified vector purity as a key determinant of vector potency *in vivo* [127,128]. Increasing vector purity and potency is predicted to reduce the risk of deleterious immune responses after administration of recombinant AAV to human subjects. Standard purification of AAV vectors depends on multiple rounds of CsCl ultracentrifugation. However, these gradients are limited in terms of their loading capacity and prolonged exposure to CsCl has been reported to compromise AAV vector potency. Large-scale purification of AAV has been based on ion-exchange chromatography, hydrophobic interaction, and affinity purification methods, resulting in increased purity and yields. However, these methods are generally serotype dependent and lack the ability to discern between fully functional AAV particles and empty capsids. In contrast, density centrifugation using iodixanol gradients effectively separates infectious particles from empty capsids [127]. 

## 9. Conclusions and Future Perspectives

Initial gene transfer studies heralded great promise for this novel class of therapeutic agents. However, initial progress was hyped and too little effort was oriented towards understanding basic aspects of vector biology and host immunology, resulting in a general failure of the field to deliver clinically applicable gene transfer strategies. Nevertheless, under the impulse of unremitting efforts by many researchers, the past decade has seen great progress in the detailed characterization of many facets of gene transfer vectors. To date, AAV represent the most promising gene transfer vehicle and studies have shown great efficiency in various animal models. With the advent of large scale AAV production, these advances have recently finally been extrapolated to humans, resulting in the successful completion of numerous phase I clinical trials for mendelian disorders including Leber's congenital amaurosis and hemophilia B. These studies constitute an important landmark underscoring the vast improvements of gene transfer technologies over the past decade. In light of these achievements, the recent positive assessment by the European Medicines Agency's Committee for Medicinal Products for Human Use (CHMP) of the marketing authorization for the first gene transfer product in Europe (alipogene tiparvovec; Glybera^®^) highlights an important paradigm shift by regulatory agencies, as well as the biotechnological entrepreneurs and investors. However, despite this significant progress, clinical experience with gene transfer technologies remains very limited and many unanswered questions warrant further study. Therefore, the field should continue to progress as it has over the past decade, cautiously and diligently.
